# Anticipating, measuring, and minimizing MEMS mirror scan error to improve laser scanning microscopy's speed and accuracy

**DOI:** 10.1371/journal.pone.0185849

**Published:** 2017-10-03

**Authors:** John P. Giannini, Andrew G. York, Hari Shroff

**Affiliations:** 1 Section on High Resolution Optical Imaging, National Institute of Biomedical Imaging and Bioengineering, National Institutes of Health, Bethesda, Maryland, United States of America; 2 Biophysics Program, University of Maryland, College Park, Maryland, United States of America; 3 Calico Life Sciences LLC, South San Francisco, California, United States of America; Pennsylvania State Hershey College of Medicine, UNITED STATES

## Abstract

We describe a method to speed up microelectromechanical system (MEMS) mirror scanning by > 20x, while also improving scan accuracy. We use Landweber deconvolution to determine an input voltage which would produce a desired output, based on the measured MEMS impulse response. Since the MEMS is weakly nonlinear, the observed behavior deviates from expectations, and we iteratively improve our input to minimize this deviation. This allows customizable MEMS angle vs. time with <1% deviation from the desired scan pattern. We demonstrate our technique by optimizing a point scanning microscope’s raster patterns to image mammal submandibular gland and pollen at ~10 frames/s.

## Introduction

Many applications in biomedical microscopy require imaging with high spatiotemporal resolution. Imaging techniques now provide spatial resolution at or surpassing the diffraction limit, and temporal resolution down to the sub-millisecond level. The ability to perform accurate, controllable, high speed scanning is fundamental to most of these methods. For example, imaging at frame rates of tens to hundreds of Hz is necessary to capture functional dynamics in neural tissue [1, 2]. In rescan confocal microscopy [3], a super-resolution imaging technique, accurate synchronization of excitation and emission scanning is essential in order to extract sub-diffractive spatial information from the sample. Similarly, in light sheet microscopy, strict synchronization of the illumination beam with the camera’s rolling shutter enables real-time rejection of out-of-focus light [4]. Regardless of the particular application, temporal resolution is often limited by choice of scanning hardware and scanning mechanism.

Many modern laser scanning microscopy techniques use galvanometer-controlled mirrors to move the illumination beam relative to the sample [5]. Larger (~5mm) non-resonant galvanometers, traditionally used for slow scanning and step-stop operation, are very accurate and feature low settling times (100–300 μs) for small motions. Galvanometer-controlled mirrors are fundamentally speed limited by their size, inertia, and the requirement to slow down and reverse direction. For most moderate fields of view (FOVs), these mirror scanning systems have traditionally limited imaging frame rates to several Hz [6]. Resonant galvanometer-controlled mirrors are capable of much higher speeds than conventional galvanometric scanners, on the order of ~10^4^ lines per second, enabling video rate or faster frame rates [7]. However, the fixed-frequency sinusoidal motion of resonant scanners impedes imaging at variable rates or random-access scanning (where only discrete portions of the field of view are scanned [8]). Also, resonant scanning is not performed at constant velocity, so illumination dwell time is not constant, resulting in non-uniform detection sensitivity across the region of interest.

To a lesser extent, rotating polygonal mirrors and acousto-optic deflectors (AODs) are also used in laser scanning applications, and they present their own advantages and drawbacks [5]. Polygonal mirrors enable rapid scanning (1–4 kHz line rates) with adjustable speed. In contrast to resonant mirrors, the angular range is limited by the number of facets, effectively fixing the field of view. Another disadvantage of polygonal mirrors is that the rotation axis is distant from the mirror face, meaning over the scan period of each mirror face, the axial path length varies during a scan. AODs use radio frequency sound waves to create a tunable diffraction grating that is used to control laser beam output angle. The absence of moving mechanical parts allows AODs to scan at very rapid speeds (approaching line rates of 1 MHz), and they allow random-access scanning, which enables frame rates of > 1kHz [8]. Drawbacks of AODs include their relatively small scan range (<4 degrees) and high dispersion. Such dispersion leads to transmission losses and wavefront distortions that can be compensated to some degree with additional hardware [8, 9]. Because AODs do not transmit emitted light from the sample efficiently, they are not typically used for rescanning applications.

Improvements in microelectromechanical systems (MEMS) scanner technologies have permitted their increasing use in high speed beam steering. MEMS mirror scanners are available in a range of sizes (~0.5–5 mm), and can access moderate angles (> +/- 10 degrees optical) at high speeds (300 Hz to 6 kHz line rates). MEMS scanners have proven useful for applications in optical coherence tomography [10, 11], confocal reflectance microscopy [12], two-photon microscopy [13], microendoscopy [14], and light-sheet microscopy [15].

MEMS mirrors, galvanometer-controlled mirrors, and polygonal mirrors are all typically driven by either an open-loop or closed-loop control system. In an open-loop system, the output of the system does not inform or improve the control action at all. Many galvanometers and polygonal mirrors provide a built-in means for sensing the position of the mirror either electronically or optically. This allows for closed-loop control, where the position sensing corrects and improves the control action. An example of a closed-loop feedback mechanism is proportional, integral, and derivative (PID) control [16], which incorporates and predicts past, present, and future sources of error to improve performance. Until recently [17], MEMS mirrors have been typically controlled with an open-loop system.

Here we present a method to “close the loop” for a MEMS device without built-in position sensing, using a feedback mechanism comparable to PID control. Our improved control algorithm enables faster, more precise scanning than previously possible. First, we examine the performance of a MEMS scanner with traditional control methods. Second, we account for the device’s impulse response in the control algorithm and demonstrate improved scan accuracy and performance. Third, we show the accuracy of the algorithm can be further improved by iteratively measuring and correcting for the observed behavior of the MEMS mirror. Finally, we demonstrate applications of our control algorithm by using it to optimize fast raster patterns and perform point scanning microscopy on biological test specimens.

## Materials and methods

**Fig 1:**
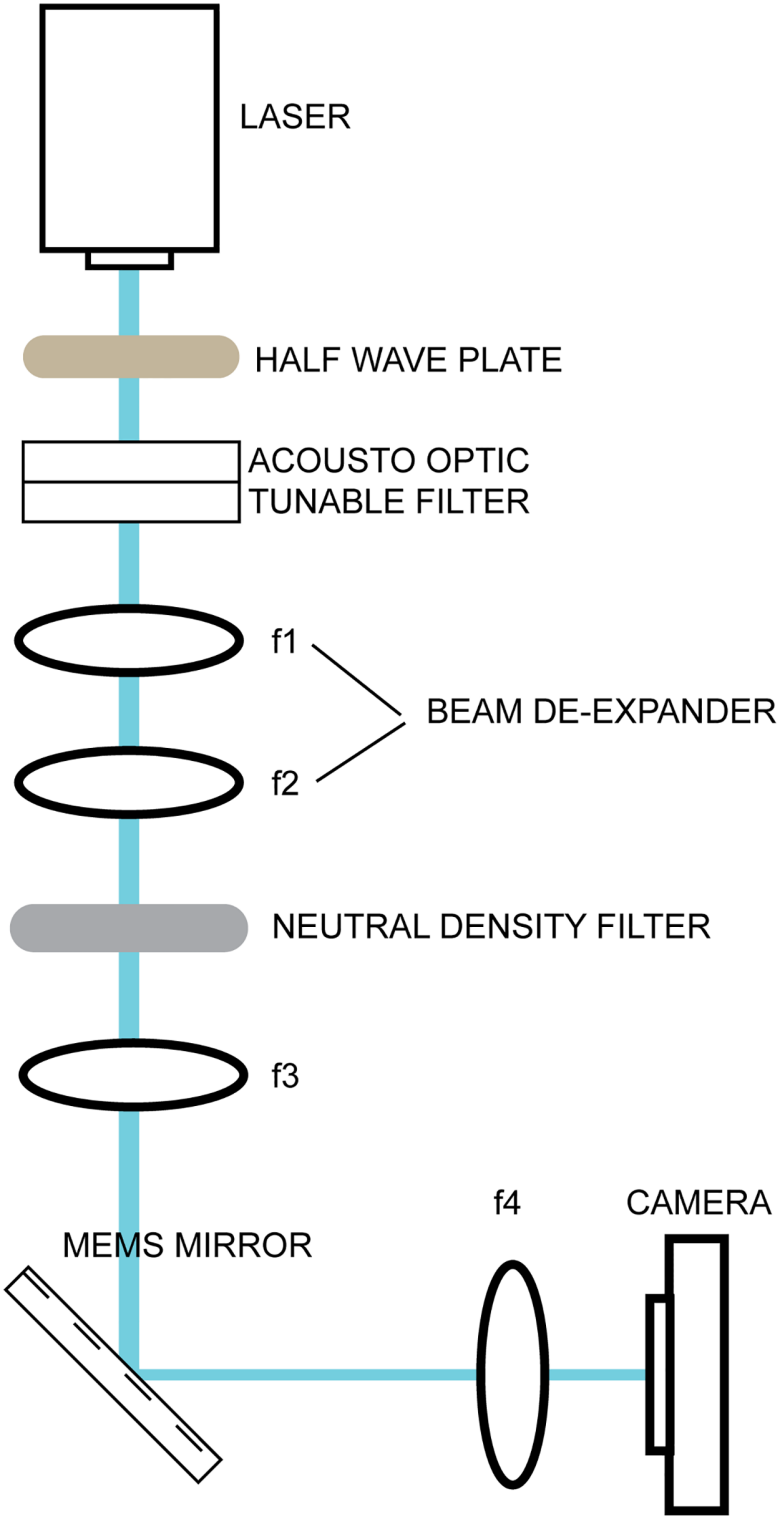
Laser scanning test rig. Test rig for characterizing mirror performance.

We began our investigation using a 1.2 mm MEMS mirror (Mirrorcle, A1B2.5-1200AL-DIP24-A/TP), with an angular range of +/ 9.2 degrees optical, which allowed for a large field of view, and a resonant frequency of 3.25 kHz, which facilitated rapid scanning. In order to characterize mirror performance we built a test rig (Fig 1), which scanned a laser beam across a camera chip.

A 200 mW, 488-nm laser was used for illumination. The laser was passed through an acousto-optical tunable filter (AOTF) for fast shuttering and dynamic intensity control. The intensity of the laser through the AOTF was maximized by tuning the rotation of a half-wave plate placed in front of the AOTF. After the AOTF, the beam was contracted by 7/8 with a telescope. The lenses in this telescope, f1 and f2, were separated by the sum of their focal lengths to preserve beam collimation. Post-telescope, the collimated beam was then passed through a reflective neutral density (ND) filter to further attenuate intensity. The attenuated beam was focused onto the pivot point of a 1.2 mm diameter MEMS mirror (placed in a DIP24 package and mount) by placing lens f3 one focal length away from the MEMS mirror. Because lens f4 is also placed one focal length away from both the MEMS mirror and the scientific-grade complementary metal-oxide semiconductor camera, scanning the angle of the mirror changes the position of the beam on the camera. This allows us to measure the mirror's approximate angle vs. time in response to an input voltage vs. time by taking a series of images and observing the resulting laser positions on the camera. Voltages were issued from a PC via an analog out card, and a bias differential quad-channel (BDQ) amplifier was used to amplify voltage signals provided to the MEMS mirror. A complete components list can be found in S1 File.

**Fig 2:**
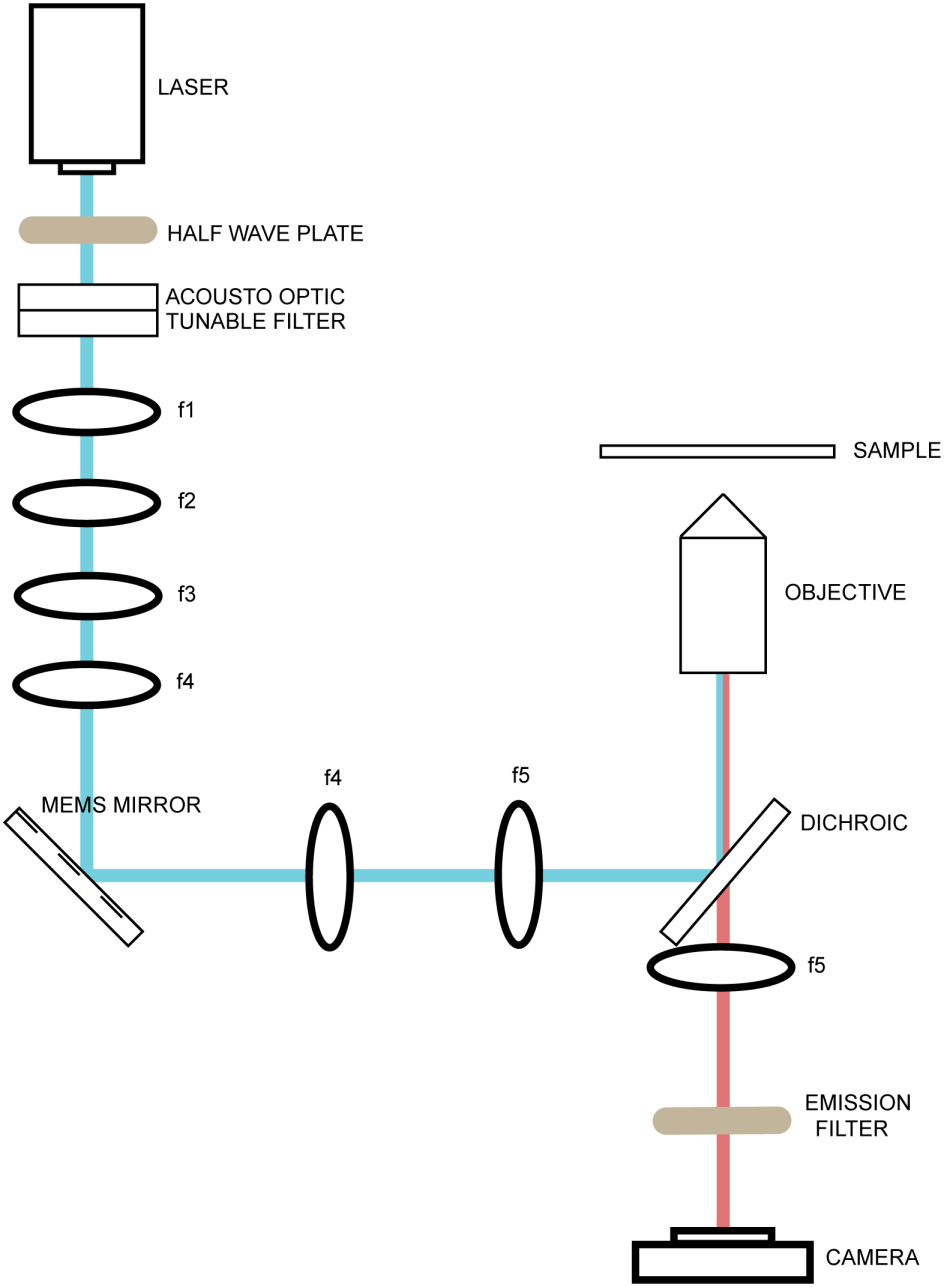
Point scanning microscope test rig. Test rig for point scanning fluorescence microscopy.

To demonstrate the value of our waveform optimization method for imaging, we also built a simple point scanning fluorescence microscope (Fig 2).

As before, a 200 mW, 488-nm laser was used for illumination and fast shuttering and dynamic intensity control used achieved with an AOTF. After the AOTF, the beam was contracted by two consecutive telescopes. The first telescope contracted the beam by 7/8, and the second by 8/25. The beam was then scanned by a 1.2 mm diameter MEMS mirror placed in a DIP24 package. The MEMS was imaged to the back focal plane (BFP) of the objective with a telescope and a dichroic beamsplitter. Fluorescence from the sample was collected with the same objective, transmitted through the dichroic beamsplitter, and passed through an identical tube lens onto a scientific-grade complementary metal-oxide semiconductor camera. Excitation light was removed by an emission filter placed before the camera. Voltages were issued from a PC via an analog out card, and a bias differential quad-channel (BDQ) amplifier was used to amplify voltage signals provided to the MEMS mirror. A complete components list can be found in S2 File.

## Results

In order to successfully gauge the mirror’s capabilities, we needed the ability to accurately measure the mirror's angle vs. time in response to an input voltage vs. time. To enable this measurement, we built the test rig shown in Fig 1. Because this rig enables us convert position on the camera to mirror angle, if we strobe the laser light, we can use the position of the laser on the camera to determine the mirror angle at the time of the strobe. To completely measure the angle vs. time response of an extended input voltage, it is necessary to collect many of these measurements. This was accomplished by repeating the input voltage while strobing the laser once per input, varying the delay of the illumination strobe to map out the mirror’s response. Between successive strobes, we allowed for a cooldown period (~0.5 seconds) to ensure that each measurement was independent of the last.

**Fig 3:**
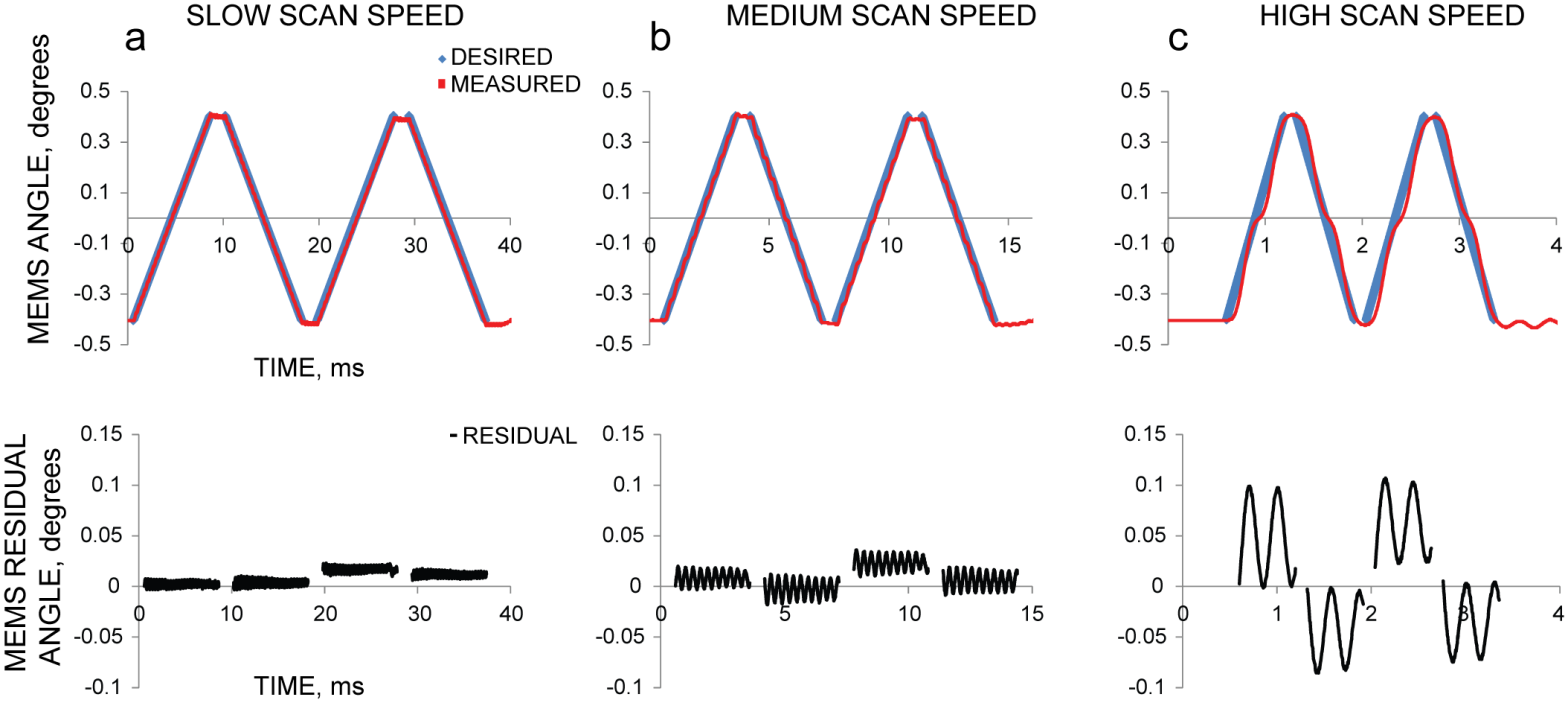
Naïve input voltages become inaccurate for high-speed MEMS operation. At slow scan speeds (left column (a), 8 ms/sweep, 1.6 ms/turnaround between forward and backward sweeps), using an input directly proportional to the desired result produces a reasonably accurate output. However, as the speed of the scan increases (middle Column (b), 3 ms/sweep, 0.6 ms/turnaround; right Column (c), 0.6 ms/sweep, 0.12 ms/turnaround), residual error between the desired and achieved patterns also increase. At speeds desirable for many scanning applications (right column (c)), the scan pattern is unusable. The top row compares the desired scan pattern with the measured result. The bottom row shows the residual error (between the desired and achieved pattern).

To characterize the mirror response at different speeds, we devised several similar simple scan patterns, consisting of four constant-velocity sweeps (two cycles over ~0.85 degrees), that differed only in relative speed (Fig 3). At slow scan speeds (3–8 ms/sweep, Fig 3a and 3b), an approximation of the mirror impulse response as a delta function produced fairly accurate results, because the settling speed of the mirror was fast compared to the desired scan frequency; the angular error (difference between desired and actual response of the MEMS mirror) was < 10%. The mirror could thus be controlled by a “naïve” waveform, directly proportional to the desired scan pattern (S1a Fig). However, as the speed and complexity of the desired scan pattern increased (0.6 ms/sweep, Fig 3c), the accuracy of this method degraded to ~25% peak error. Fast, high-accuracy operation was not possible without accounting for the mirror’s impulse response.

We used our strobe-based measurement system to characterize the mirror’s impulse response, finding that it was modeled well by an exponentially-decaying sinusoid. With the desired scan pattern and a model of the mirror impulse response, we used a modified Landweber deconvolution [18] to solve the inverse problem of what voltage waveform needed to be sent to the mirror to produce the desired output scan pattern more accurately:

$$V(n+1)=V(n)+\lambda H^{T}(H(V(n))-D)$$ (1)

In Eq (1), V(n) represents the current input voltage waveform at iteration n, λ represents the relaxation factor, **H**^T^ represents the transpose operator, **H** represents the forward operator, and D represents the desired (known) output.

The iteration has two major components: a forward operator, **H**, and a transpose operator, **H**^T^. The forward operator consists of a blurring step, where the input is convolved with the mirror impulse response to produce an expected result, and a cropping step, where the expected result is cropped to only account for important scan regions. After cropping and blurring the input voltage to produce the expected output, we compare the result to the desired (and similarly cropped) result to produce a residual. The transpose operator, **H**^T^, consists of a crop transpose step, where the residual is zero padded to restore the length of the original input voltages, and a blur transpose step, where the residual is convolved with the time-reversed impulse response. The transpose operator assigns blame to the input for disagreements between the expected response and the desired response, producing a correction voltage. This correction voltage is added to the original input to produce an improved input voltage (S1b Fig). For our waveforms, we found that the algorithm usually converged with n ~ = 5000 iterations using λ = 0.004 (30–60s on our acquisition computer). Setting higher values for λ can speed convergence but also occasionally causes divergence.

**Fig 4:**
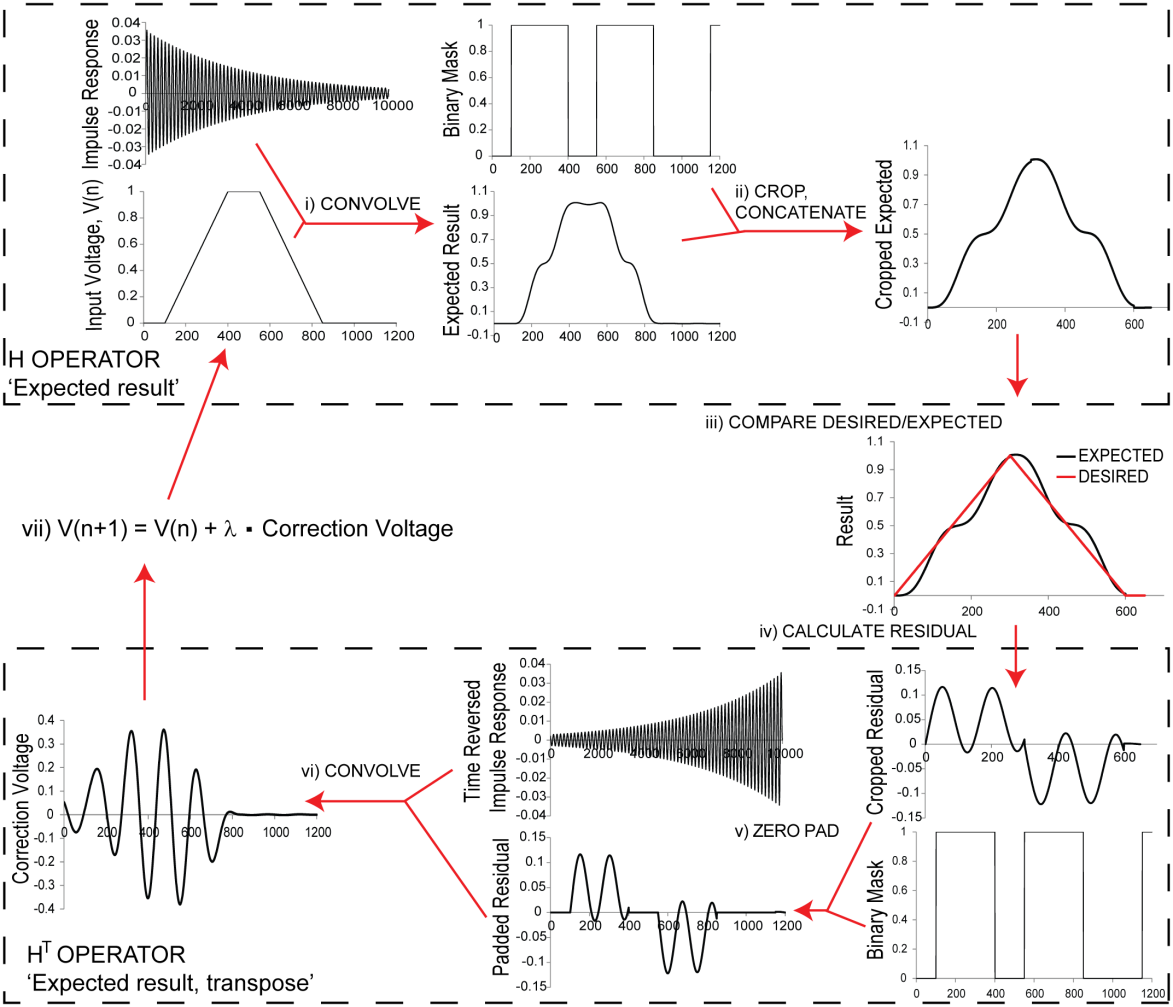
Landweber-based deconvolution optimization. After measuring each MEMS mirror’s response to an impulse, iterative deconvolution can be used to determine a set of input voltages that will more closely produce the desired output scan pattern. We use a Landweber iteration to solve this inverse problem. The iteration has two major components: A forward operator (**H**), which takes a desired input and produces the expected result after convolution with the MEMS mirror impulse response, and a transpose operator (**H**^T^), which assigns blame to the input for disagreements between the expected response and the desired response. The forward operator consists of: **i**) a blurring step, in which the current set of input voltages V(n) is convolved with the impulse response, and **ii**) a cropping step, in which only the results in the scan regions are considered. Cropping is performed because constraining the procedure to defined scan regions allows for higher accuracy in these regions (see S2 Fig), and because it is difficult to define exactly what the "desired" result is in undefined regions. Practically we carry out the cropping operation by comparing the blurred voltages with a binary mask (defining the constrained scan regions), and concatenating the resulting masked regions. In addition to the constrained scan regions, there is a small constrained region at the end of each waveform to ensure that the mirror settles quickly to its original position. After producing the cropped, blurred voltages, we compare **iii**) the result to the desired (and similarly cropped) result to produce a residual **iv**). The transpose operator consists of a ‘crop transpose’ step **v**), where the residual is again compared to the binary mask and zero padded to restore the length of the original input voltages; and a ‘blur transpose’ step **vi**), where the padded residual is convolved with the time-reversed impulse response. This produces a ‘correction voltage’ which is multiplied by a relaxation factor λ and added to the original input voltage V(n) to produce a corrected input voltage V(n + 1), **vii**). Empirically, we find that λ = 0.004 and n = 5,000 iterations produce good results. For clarity, we have omitted units on the vertical (proportional to voltage) and horizontal (time or index) axes in all graphs.

Because many scanning applications have regions where accuracy is unimportant (e.g. flyback regions), we modified the Landweber iteration (Fig 4, S3 File) to use only important regions for optimization. This modification makes it possible to achieve higher accuracy in important areas of the scan pattern by neglecting unimportant areas (S2 Fig).

The computational burden associated with repeated convolution operations makes it necessary to optimize large waveforms piecewise. The long tail of the mirror’s impulse response means that changes at the beginning of a large waveform influence later portions of the waveform, so waveforms were all optimized piecewise from beginning to end. Also, for all of our optimized waveforms, we added a short constrained region at the end of each waveform to ensure that the mirror settled properly to its original position.

**Fig 5:**
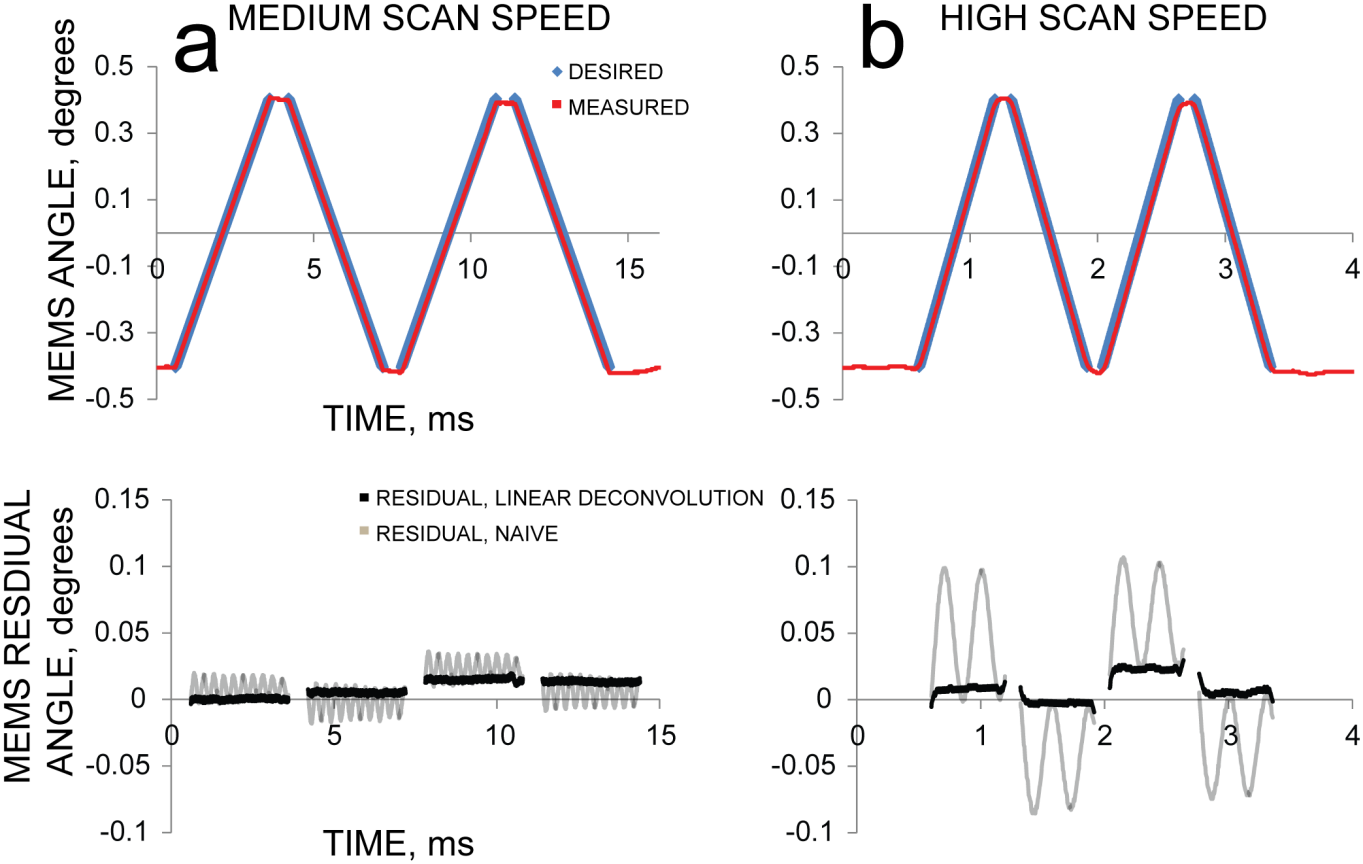
Using linear deconvolution to determine input voltage improves scan accuracy compared to naïve voltages. The response of the mirror at high speed can be greatly improved by using Landweber deconvolution to determine the input voltage. However, nonlinearities in the mirror response still produce a non-trivial residual. As in Fig 3, the top row shows the desired scan pattern and measured result, and the bottom row shows the residual. Compare left (a) and right (b) columns to Fig 3b and 3c middle and right columns, respectively; residual data from Fig 3b and 3c is shown here in gray.

Our modified Landweber iteration greatly improved the accuracy of the achieved scan pattern (Fig 5). Using the optimization algorithm reduced the maximum residual between desired and achieved pattern two-fold for the medium scan speed (3 ms/sweep, Fig 5a) and four-fold for the high scan speed (0.6 ms/sweep, Fig 5b). While this improvement is significant, the residual was worse for patterns at higher speeds. We suspected that the residual differences were due to nonlinearities in the mirror’s impulse response that were not accounted for by our linear deconvolution method. To address this issue, we developed another iterative process to reduce this residual by further improving the input voltage. We started with the same Landweber-based algorithm to produce an input voltage, V(n), that we expected to produce the desired scan pattern, D. Next, we measured the mirror’s actual response, and calculated the residual between desired and measured scan pattern. Then we set D to the measured residual, re-ran our Landweber algorithm to calculate a correction voltage optimized to produce the residual, and subtracted this voltage from the previous input voltage, producing an improved voltage waveform with lower residuals.

**Fig 6:**
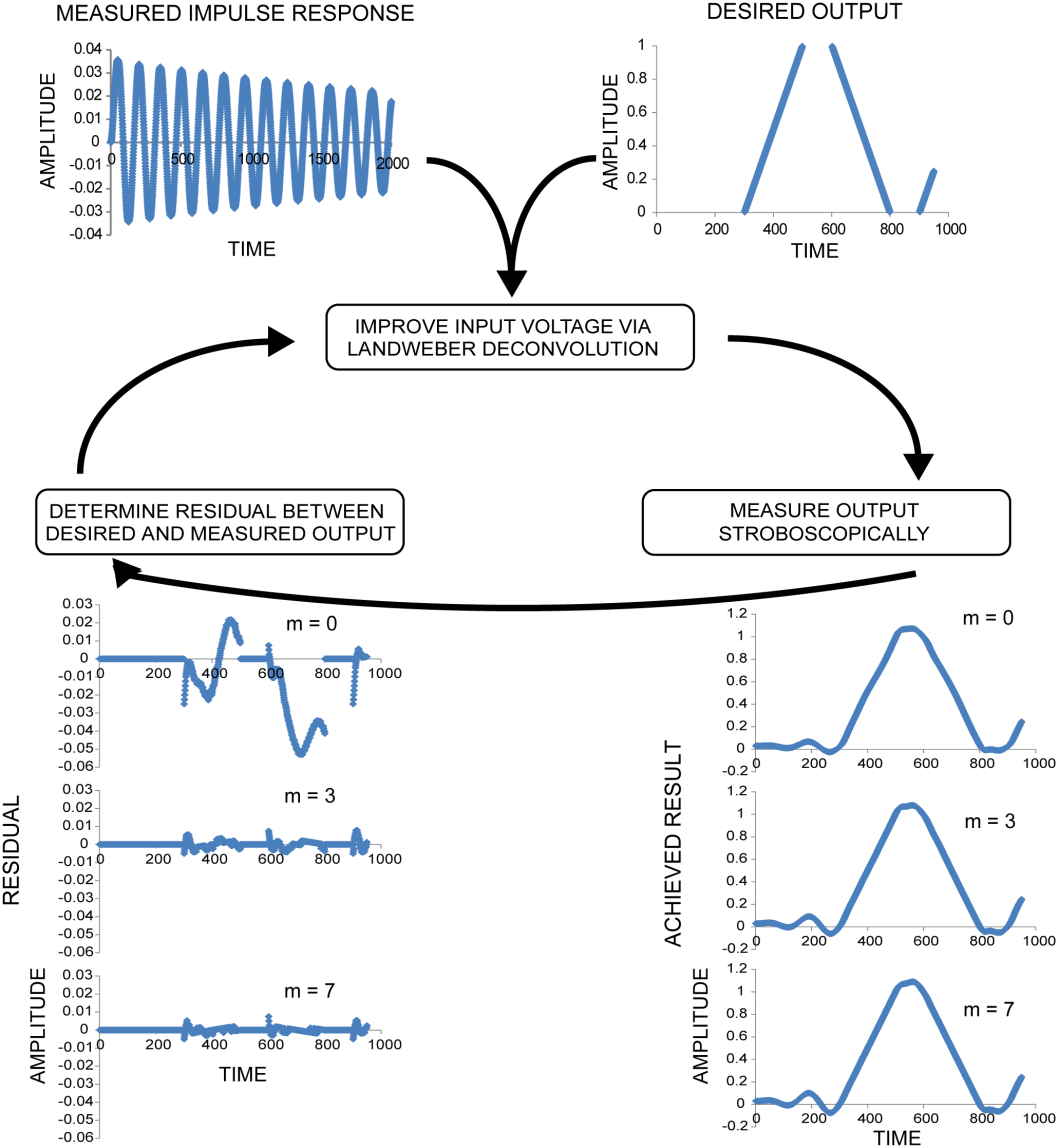
Overview of iterative, measurement-based deconvolution method for precise MEMS mirror control. Given the measured impulse response (top left) and desired output (top right), deconvolution (middle) provides an input that produces a measured output that approximates the desired output (right, m = 0). Nonlinearities in the mirror’s response lead to a difference (residual, left, m = 0) between expected and measured responses, especially at high speeds. However, the deconvolution algorithm can incorporate the measured residual, computing a modified input that reduces the residual error. Repeating this procedure over a few measurement cycles (examples shown after 3, 7 iterations) dramatically lowers the residual, producing the desired result with high accuracy.

**Fig 7:**
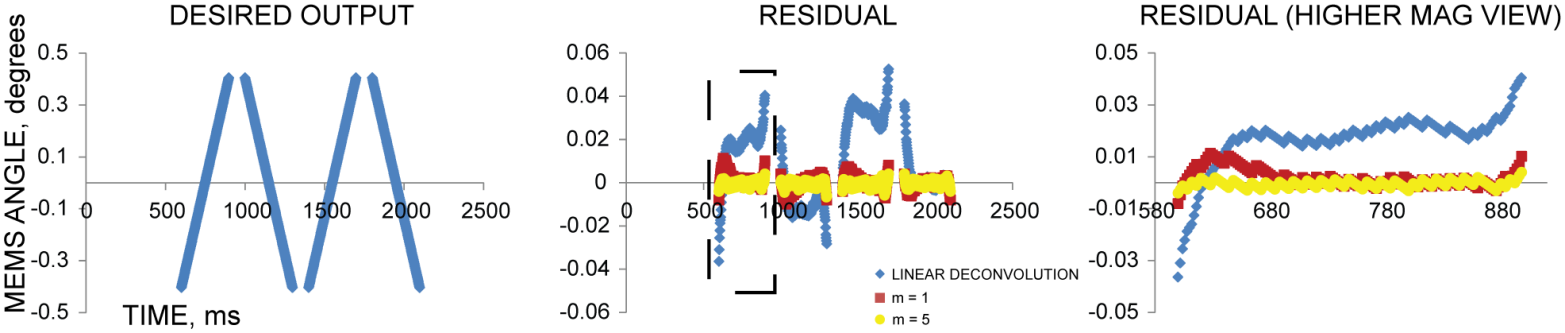
Response of the MEMS mirror after using iterative, measurement-based deconvolution. The response of the mirror converges to the desired scan pattern (left) by iteratively measuring and incorporating the residual (difference between the desired and achieved pattern) into the deconvolution method. The residual in constrained scan regions is used to compute a set of correction voltages that will cancel out the remaining residual. These correction voltages, produced by deconvolution after each measurement cycle, eventually lower the residual (middle, right) to within 1% of the desired result. Shown is a scan pattern with 300 μs /sweep and 100 μs /turnaround, with linear deconvolution (blue diamond), m = 1 iteration (red square) and m = 5 iterations (yellow circle).

By repeating this outer iteration based on measured residuals (Fig 6, S3 File), we obtained a nearly optimal set of input voltages for a desired scan pattern (S1c Fig). We found that after one iteration of the optimization algorithm (m = 0), the error was usually within +/- 5% of the desired result. After a few iterations (m = 3), the error was within +/- 1% of the desired result, and after more iterations (m = 5), the results were only marginally improved (Fig 7). Including measurement and computation time, optimization of these test waveforms (m = 5 iterations) was achieved in ~1 hour.

**Fig 8:**
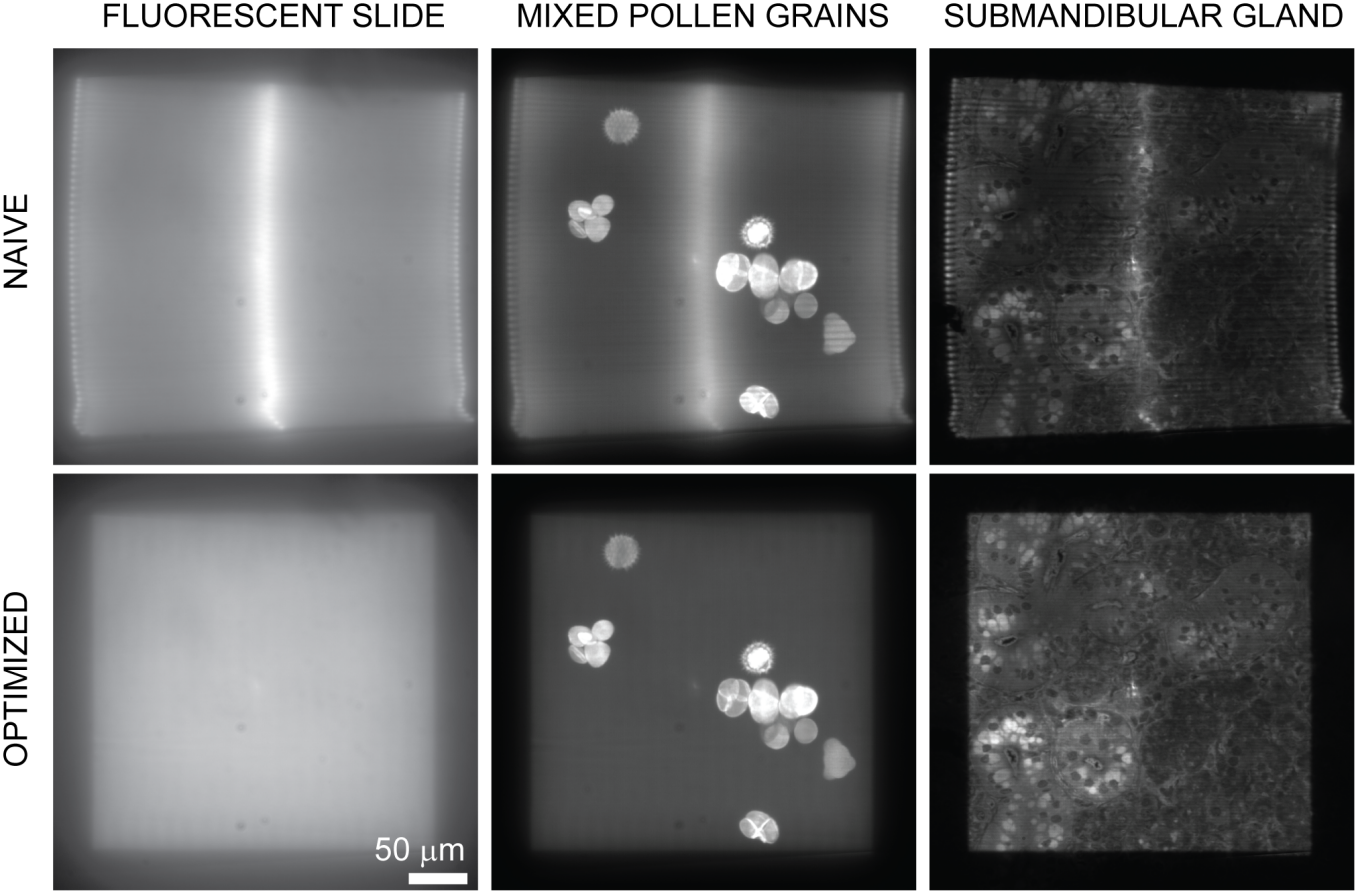
Optimized illumination scanning improves fluorescence image quality. Images of a plastic fluorescence slide (left column), mixed pollen grains (middle column), and submandibular gland (right column) were acquired by scanning the excitation focus across the field of view in a raster pattern and recording the fluorescence on a camera. Results obtained with a naïve raster waveform (top row) are compared to the optimized waveform (bottom row). The naïve results show pronounced intensity variation across the scan (especially obvious when comparing the middle of each scan to the periphery), warping of the overall raster pattern, and obvious “lininess” within each imaging field. These artifacts are corrected when using the optimized waveform.

To demonstrate the value of our waveform optimization method for point scanning fluorescence imaging, we used the test rig described in Fig 2. We performed raster scanning using both a naïve waveform and a waveform optimized using our measurement-based deconvolution method (Fig 8). In both cases, the raster pattern was made up of a 111 lines scanned at a speed of 600 μs per line with 300 μs turnaround. During the turnaround time, the slow axis line shift of the raster pattern is performed. For the optimized waveform, the optimization was performed by constraining both dimensions during the scan and leaving both unconstrained during the turnaround. This pattern enabled image collection at just under 10 Hz. We collected images of several test samples, including a plastic fluorescent slide (Fig 8, left column), mixed pollen grains (Fig 8, middle column, Carolina 30–4264), and submandibular gland (Fig 8, right column, Carolina 31–4932), to compare the image quality obtained with each waveform. The naïve results in the top row show intensity variation across the scan, especially in the center and along the vertical edges. Because the naïve waveform does not incorporate the impulse response of the MEMS mirror, the MEMS mirror does not scan the illumination at a uniform speed. We thus attribute the high intensity vertical line areas in the top row images to higher illumination dwell times arising from the inconsistent scan speed. Additional artifacts produced by the naïve waveform include “lininess” at the image edges and warping of the edges. The optimized waveform removes these artifacts, restoring image quality across the whole field of view for each sample. Note that scan errors only affect the uniformity of illumination for our simple point-scanning microscope, but for a confocal microscope (especially rescan confocal (3)]), the scan errors shown in the top row would cause substantial distortion in both the apparent brightness and apparent position of fluorophores in the sample.

## Discussion and conclusions

We present a novel waveform optimization algorithm for improved MEMS mirror control. While at slow scan speeds, (8 ms/sweep, 1.6 ms/turnaround, Fig 3a), approximating the mirror’s impulse response with a delta function produced fairly accurate results (<4% peak error), a different control method is necessary for faster and more accurate scanning. Our algorithm incorporates the mirror’s impulse response and uses Landweber-based deconvolution to generate input voltages waveforms that correctly produce the desired output. By iteratively applying the algorithm and incorporating measurements of the resulting scan pattern, residual errors for much faster scan speeds of 300 μs/sweep and 100 μs/turnaround (Fig 7) can be corrected to less than 1% of the desired result. This represents a 24x speed improvement while also improving peak error from <4% to <1%. Although the optimization process is currently time-consuming, it only has to be performed once, unless the hardware changes. As we demonstrate, the algorithm enables accurate image formation at high speed, avoiding serious artifacts that would result if input voltages were applied without the algorithm (Fig 8).

We envision many applications of our approach beyond the MEMS based raster image scanning we demonstrate. In future work, we hope to show its applicability in rescan microscopy (3)] to increase speed while maintaining high accuracy. Our iterative control feedback algorithm can also be applied to non-MEMS hardware, essentially any repeatable, nearly-linear system with a measurable impulse response. We have, for example, explored using the algorithm to increase the scan capabilities of a piezoelectric actuator plate. Future improvements could improve the algorithm’s speed and accuracy. Measuring the mirror’s response to input voltage waveforms currently accounts for approximately 83% of the total optimization time, while computation accounts for the remaining 17%. Computation time could be marginally improved with more computing power, but the strobe-based characterization system we use, while accurate and comprehensive, is currently rate limiting. While it is possible to use strobe-based characterization more sparsely and interpolate missing results, this lowers the accuracy of the measurements. A more efficient scheme, such as one that strobes multiple times per measurement or incorporates illumination intensity variation, would enable an accurate system characterization with fewer measurements. Fast, reliable position sensing for MEMS mirrors could be combined with our algorithm to effectively eliminate the rate-limiting measurement of the system’s response. In this scenario, our algorithm would function as an alternative to PID control.

## Supporting information

S1 Fig Input voltage comparison. Example input voltages to MEMS mirror, comparing naïve input (i.e. proportional to desired scan pattern, a), the input predicted by linear deconvolution (b), and the input calculated by 6 iterations of our iterative, measurement-based deconvolution algorithm (c). The desired scan pattern has 300 μs /sweep and 100 μs /turnaround.
(TIF)

S2 Fig Masking the residual in unimportant areas. Most scan patterns contain regions in which accuracy is irrelevant. For instance, the turnaround or flyback regions in a raster pattern need not be accurate, as no data will be collected during this time. Furthermore, it is often difficult to define exactly what the "desired" result is in undefined regions. For the sake of this demonstration, we assume the turnaround regions, not depicted in the desired output, are stationary pauses. It is possible to achieve higher accuracy in important areas of the scan pattern if unimportant areas are neglected. This is achieved by masking (setting equal to zero) the residual in these unimportant areas, and only incorporating the residual in important areas (unimportant regions are not plotted) when performing the iterative deconvolution algorithm. In this example the desired scan pattern (left), a higher magnification view (middle, corresponding to dashed box at left) and residual (right) are shown, for a pattern with 180 μs/sweep and 60 μs/turnaround. In the residual plot, two results are shown: the red line is the residual result when only the scan region (indicated by the dashed box) is unmasked, the blue line is the residual without masking (i.e. the entire pattern is optimized). Within and especially towards the edges of the important region, the masked optimization is more accurate.
(TIF)

S1 File Laser scanning test rig components list. (DOCX)

S2 File Point scanning microscope test rig components list. (DOCX)

S3 File Optimization pseudo-code. Pseudo-Code to communicate the important portions of our algorithm. While it will not run, it strongly resembles our actual code and is hopefully substantially more readable.
(PY) Click here for additional data file.
