# Supplementary figures and images for: Anticipating, measuring, and minimizing MEMS mirror scan error to improve laser scanning microscopy's speed and accuracy
Source: PLoS One. 2017 Oct 3;12(10):e0185849. doi: 10.1371/journal.pone.0185849 (PMC5626505; doi:10.1371/journal.pone.0185849)

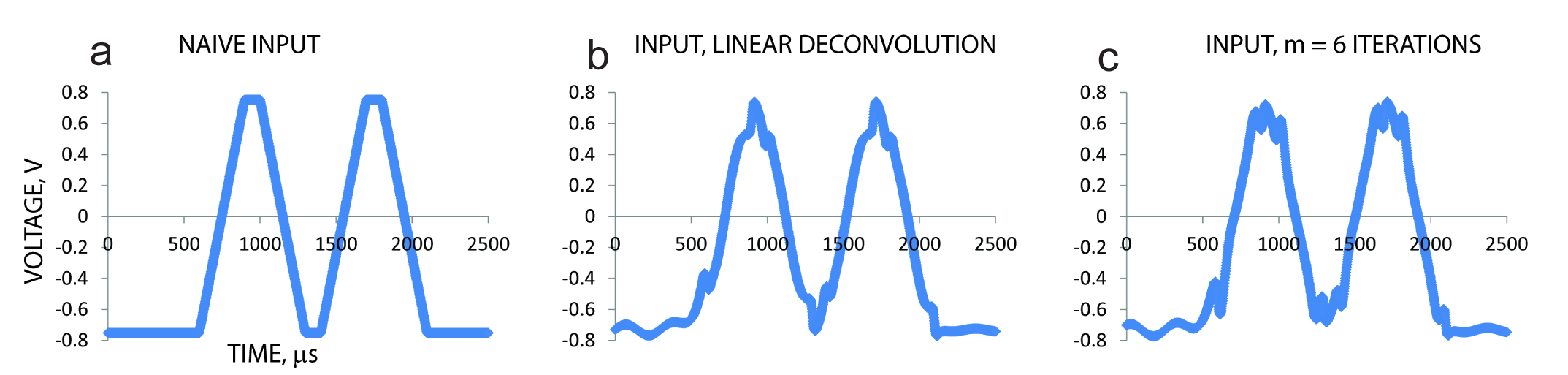

Supplement: S1 Fig — Example input voltages to MEMS mirror, comparing naïve input (i.e. proportional to desired scan pattern, a), the input predicted by linear deconvolution (b), and the input calculated by 6 iterations of our iterative, measurement-based deconvolution algorithm (c). The desired scan pattern has 300 μs /sweep and 100 μs /turnaround. (TIF) [file pone.0185849.s001.tif]

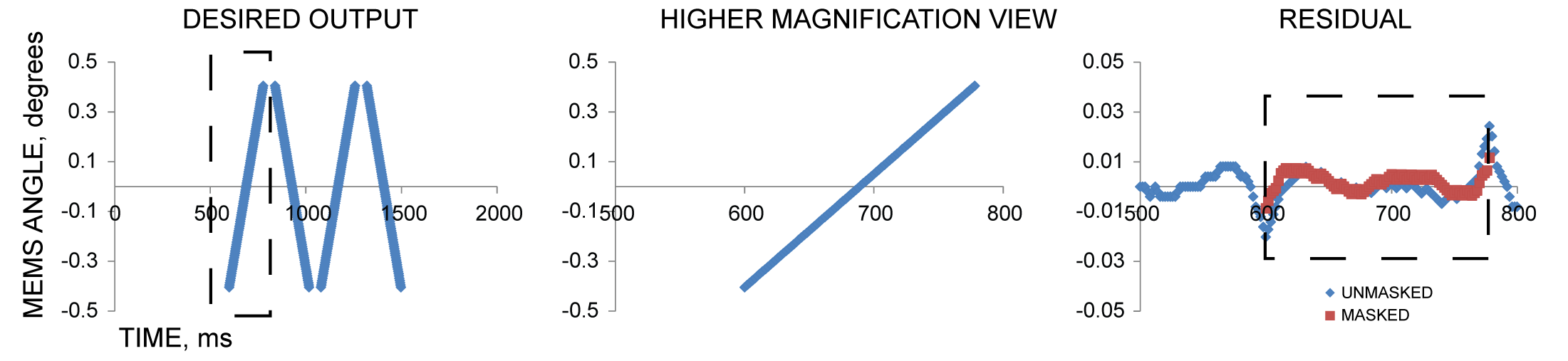

Supplement: S2 Fig — Most scan patterns contain regions in which accuracy is irrelevant. For instance, the turnaround or flyback regions in a raster pattern need not be accurate, as no data will be collected during this time. Furthermore, it is often difficult to define exactly what the "desired" result is in undefined regions. For the sake of this demonstration, we assume the turnaround regions, not depicted in the desired output, are stationary pauses. It is possible to achieve higher accuracy in important areas of the scan pattern if unimportant areas are neglected. This is achieved by masking (setting equal to zero) the residual in these unimportant areas, and only incorporating the residual in important areas (unimportant regions are not plotted) when performing the iterative deconvolution algorithm. In this example the desired scan pattern (left), a higher magnification view (middle, corresponding to dashed box at left) and residual (right) are shown, for a pattern with 180 μs/sweep and 60 μs/turnaround. In the residual plot, two results are shown: the red line is the residual result when only the scan region (indicated by the dashed box) is unmasked, the blue line is the residual without masking (i.e. the entire pattern is optimized). Within and especially towards the edges of the important region, the masked optimization is more accurate. (TIF) [file pone.0185849.s002.tif]
