# Supplementary material for: Anticipating, measuring, and minimizing MEMS mirror scan error to improve laser scanning microscopy's speed and accuracy
Source: PLoS One. 2017 Oct 3;12(10):e0185849. doi: 10.1371/journal.pone.0185849 (PMC5626505; doi:10.1371/journal.pone.0185849)
Supplement: S1 File — (DOCX) [file pone.0185849.s003.docx]

S1 Laser Scanning Test Rig Components List

- Coherent Sapphire 200 mW, 488 nm laser
- AA Optoelectronic Acousto-optical tunable filter (AOTF, Quanta Tech, AOTFnC-400.650-TN)
- Thorlabs, half-wave plate, WPH10M-488
- Thorlabs, f1 = 40 mm achromatic doublet, AC254-040-A-ML
- Thorlabs, f2 = 35 mm achromatic doublet, AC254-035-A-ML
- Edmund Optics, 1.0 OD, 30935
- Thorlabs, f3 = 75 mm achromatic doublet, AC254-075-A-ML
- Mirrorcle Technologies, Gimbal-less Two Axis MEMS Mirrors, angular range +/- 4.6 degrees mechanical, A1B2.5-1200AL-DIP24-A/TP
- Mirrorcle Technologies, DIP24 Packaging MINI-DIP24-5.x-MNT
- Thorlabs, f4 = 40 mm triplet, TRH254-040-A-ML
- PCO-TECH, pco.edge 5.5
- Mirrorcle Technologies, BDQ amplifier MEMS Driver DR-11-033-01
- @XI Custom Workstation
- National Instruments, Analog out card, PXI-6733
